# The role of primary emotions in separation-individuation during emerging adulthood

**DOI:** 10.3389/fpsyg.2026.1675495

**Published:** 2026-04-14

**Authors:** Timotej Glavač, Maja Zupančič

**Affiliations:** University of Ljubljana, Ljubljana, Slovenia

**Keywords:** affective neuroscience theory, emerging adults, parent–child relations, primary emotions, separation-individuation

## Abstract

**Introduction:**

Separation-individuation (S-I) in relation to parents is a core developmental process that unfolds during infancy/toddlerhood (primary S-I) and adolescence/ emerging adulthood (second S-I) and relates to psychological functioning across several domains. While the outcomes of this process are most commonly explained by the quality of parent–child relationships, the role of child temperament/personality is rarely considered.

**Methods:**

We examined the role of emerging adults’ primary emotions on five aspects of S-I in relation to mother and father, as captured by the Individuation Test for Emerging Adults—Short. The sample consisted of 446 emerging adults (58.7% females, 41.3% males), predominantly students.

**Results:**

Hierarchical linear regressions suggested a significant contribution of the six primary emotions (SEEKING, FEAR, ANGER, SADNESS, CARE, and PLAY) across the aspects of S-I (seeking parental support, connectedness, perceived parental intrusiveness, self-reliance, and fear of disappointing the parent), over and beyond the relevant demographic variables (age, gender, living arrangement). Differential associations showed consistent links of SEEKING with self-reliance, of CARE and low SADNESS with both support seeking and connectedness, of SADNESS with perceived parental intrusiveness, and of FEAR with fear of disappointing parents. A few inconsistent relations were likely due to somewhat different roles of mothers and fathers in the caregiving context.

**Discussion:**

Overall, our results provide new evidence supporting the idea that individual differences in primary emotional systems may play a distinct and meaningful role in affecting aspects of the S-I process in emerging adulthood.

## Introduction

Separation–individuation (S-I) is an intrapsychic process that primarily unfolds in infancy/toddlerhood and involves reciprocal processes of differentiation of the self from the caregiver (separation) that contribute to the development of a sense of autonomy (individuation) (Mahler et al., 1975). The second S-I in adolescence is focused on the restructuring of internal representations of parents as omnipotent individuals and the enhancement of inner resources that leads to a psychologically independent sense of self (Blos, 1967). In contemporary post-industrial societies, where young people postpone their responsibilities and obligations in the productive and reproductive domain of life, the process extends into emerging adulthood (18–29 years) (Beyers and Goossens, 2003; Buhl, 2008; Kins et al., 2013; Komidar et al., 2014; Lamborn and Groh, 2009; Masche, 2008).

In emerging adulthood, S-I largely involves striving for a comfortable balance between independent functioning while maintaining mutual relatedness to parents (Kins et al., 2013). Within this process the parent–child relationships become more symmetrical and reciprocal (Arnett and Tanner, 2006). Komidar et al. (2014) developed a model based on contemporary conceptualizations of S-I (e.g., Beyers and Goossens, 2003; Lamborn and Groh, 2009) and the theory of emerging adulthood (Arnett, 2000). This model was empirically supported (Komidar et al., 2014, 2016, 2021) and comprises five aspects of S-I in relation to parents. These include: *connectedness* (mutual understanding and respect, enjoyment in parental company, open communication), *support seeking* (emotional support, advice, suggestions in difficult situations or when facing important life decisions), *perceived intrusiveness* (parental intrusion into one’s privacy, overprotection, psychological control), *fear of disappointing parents* (concerns about parental reactions to one’s failure or ones actions disapproved by parents), and *self-reliance* (independent management of one’s own personal affairs). A successful resolution of the process (achievement of autonomy while maintaining relatedness to parents) has been found vital for positive psychosocial functioning, e.g., psycho-social adjustment, self-esteem, academic achievement, attainment of criteria for adulthood and life satisfaction (Delhaye et al., 2012; Kins et al., 2013; Lamborn and Groh, 2009; Zupančič et al., 2014). In contrast, unsuccessful or dysfunctional S-I is associated with difficulties in psychosocial functioning and adaptation, such as a proclivity toward anxiety and depression, insecure attachment styles and self-criticism (Holmbeck and Leake, 1999; Kins et al., 2013; Stey et al., 2014).

In line with the psychodynamic frameworks, S-I was initially conceptualized as predominantly influenced by the nature of the relationship between primary caregivers and the developing child (Blos, 1967; Mahler et al., 1975). However, the potential importance of child individual differences (e.g., temperament/personality traits) which are partly based genetically is rarely considered. Behavioral genetic research, for example, suggests that roughly half of the variance in personality traits is due to heritable genetic factors (Dochtermann et al., 2015; Knopik et al., 2016; Vukasović and Bratko, 2015), personality traits predict S-I (Zupančič and Kavčič, 2014), and individuals with certain temperament/personality dispositions are more vulnerable to adverse caregiving experiences, but thrive in optimal rearing conditions (Belsky et al., 2007).

### Personality traits, primary emotional systems and separation-individuation

Although the widely accepted model of five basic personality traits (extraversion, agreeableness, conscientiousness, neuroticism and openness; John et al., 1991; McCrae and John, 1992) shows a robust genetic foundation (Knopik et al., 2016; Vukasović and Bratko, 2015), the model lacks a theoretical framework for postulating the potential brain neuroanatomy and molecular mechanisms that underlie individual trait variations, as well as the evolutionary rationale for the five traits (Montag and Davis, 2018). An alternative model based on neurobiological studies within the Affective Neuroscience Theory (ANT) has been proposed to address some of these limitations. The ANT (Panksepp, 1991, 1998) is a robust and well-established framework to explain primary emotions from psychological, neuroscientific, and psychiatric perspectives. It encompasses seven primary emotional systems that may underlie personality traits via conditions related to the brain’s affective functions (Panksepp, 1998, 2006). These systems (SEEKING, LUST, ANGER, FEAR, SADNESS, CARE and PLAY)^1^ are phylogenetically and genetically grounded. They evolved as inherent tools for survival and for promoting overall fitness (Davis and Panksepp, 2018; Montag and Panksepp, 2017).

The primary emotional systems (henceforth primary emotions) are likely candidates for providing the neurobiological basis of the basic personality traits (Davis and Montag, 2019). Several studies using the Affective Neuroscience Personality Scales, including a meta-analysis, have suggested theoretically congruent and consistent associations of the primary emotions with four of the five basic personality traits: ANGER with neuroticism and low agreeableness, SADNESS and FEAR with neuroticism, PLAY with extraversion and SEEKING with openness (Davis and Panksepp, 2011; Marengo et al., 2021; Montag and Panksepp, 2017). Conscientiousness does not map onto the primary emotions, as this trait represents higher cortical functions, such as self-regulation capacities, which do not reside in the subcortical regions of the brain (Montag and Davis, 2018). Furthermore, LUST is not included in the respective scales, as the authors of the original measure (Davis et al., 2003) were concerned that the items related to sexual content could hinder responses and threaten the scale’s psychometric properties.

The SEEKING system is accompanied by feelings of zest, enthusiasm and curiosity (Davis and Panksepp, 2011; Panksepp, 1998). Individuals high in SEEKING are interested in novelty, have a wide range of interests and tend to explore many diverse phenomena and events. These traits also represent lower-order traits of openness (Marengo et al., 2021), which fosters greater self-reliance and independent exploration in individuals (Luyckx et al., 2012; Poredoš, 2020; Zupančič and Kavčič, 2014). The FEAR system helps individuals avoid physical danger that might lead to physical harm and potential death (Davis and Panksepp, 2011; Panksepp, 1998). Individuals with high levels of FEAR tend to interpret stimuli as threatening and perceive their environment as dangerous (Britton et al., 2011; Perkins et al., 2020; Sagliano et al., 2014). This tendency is linked to an enhanced behavioral inhibition system (Gray, 1987), characterized by high levels of anxiety and avoidance—the marker traits of neuroticism (Barlow et al., 2014; Caspi and Shiner, 2006). Neuroticism also predicts anxiety and social inhibition in interpersonal relationships (e.g., Karataş et al., 2019; Zupančič et al., 2011), such as perceived parental intrusiveness and fear of disappointing parents (Poredoš, 2020; Zupančič and Kavčič, 2014). The ANGER system enables mammals to defend themselves and their territory from hostile others (Davis and Panksepp, 2011; Panksepp, 1998). In humans, this system associates with the more broadly defined trait of agreeableness (e.g., Glavač and Zupančič, 2024; Marengo et al., 2021). Individuals low in agreeableness often experience anger and are prone to hostility, and aggressive behavior (Bettencourt et al., 2006; García-Sancho et al., 2017; Pailing et al., 2014). Likewise, low levels of agreeableness in emerging adults predicted higher levels of perceived parental intrusiveness (Poredoš, 2020; Zupančič and Kavčič, 2014).

The SADNESS system evolved as an evolutionary adaptation to maintaining proximity between the developing child and caregiver and evokes experiences of sadness and separation anxiety when the child is separated from the caregiver (Davis and Panksepp, 2011; Panksepp, 1998). In addition to anxiety, worry, stress, and guilt, a heightened tendency to experience sadness maps onto the broader trait of neuroticism (Caspi and Shiner, 2006), i.e., the trait predictive of both perceived parental intrusiveness and fear of disappointing parents in emerging adults (Zupančič and Kavčič, 2014). Similarly, the CARE system evolved in the function of maintaining caregiver-child proximity. From the perspective of the caregivers, it motivates them to care and protect their young. It also promotes social bonding and attachment (Davis and Panksepp, 2011; Panksepp, 1998). The activation of this system evokes feelings of empathy, a need for taking care of others and affection, i.e., characteristics subsumed by the basic trait of agreeableness (Marengo et al., 2021). Accordingly, agreeable individuals are likely to establish and maintain positive interpersonal relationships, including adolescent or emerging adult-parent relationships (De Haan et al., 2012; Denissen et al., 2009), seeking parental support in difficult situations and feelings of mutual connectedness with parents (Poredoš, 2020; Zupančič and Kavčič, 2014). Lastly, the PLAY system is thought to have evolved to promote social learning in young developing mammals (Davis and Panksepp, 2011; Panksepp, 1998). PLAY is accompanied by happiness, sociability and optimism (Montag and Panksepp, 2017)—the marker traits of extraversion (Caspi and Shiner, 2006; John et al., 1991; McCrae and John, 1992). Similar to agreeableness, extraversion in emerging adults has been found to predict connectedness with parents and seeking parental support when facing serious difficulties or important life decisions (Poredoš, 2020; Zupančič and Kavčič, 2014).

In addition to the nature of child–parent dynamics, personality trait differences are likely to play a significant role in the second S-I of emerging adults in relation to their parents. To our knowledge, only two studies have examined associations between the five basic personality traits and aspects of the second S-I (Poredoš, 2020; Zupančič and Kavčič, 2014). The results suggested differential associations of both extraversion and agreeableness with connectedness to parents and seeking parental support, of low agreeableness with perceived parental intrusiveness, of neuroticism with both intrusiveness and fear of disappointing parents, and of openness with self-reliance in relation to parents. Drawing upon these findings, we proposed the relevance of each of the primary emotions to the aspects of S-I in emerging adults. The primary emotions as defined within the ANT are namely proposed to represent the neurobiological substrate of the basic personality traits (Davis and Montag, 2019) and may provide a unique framework for understanding the dynamics of the S-I process, offering a perspective beyond those of traditional personality models.

### The present study

The aim of the present study was to explore the potential predictive value of individual differences in the expression of primary emotions for each of the five aspects of S-I among emerging adults. Precisely, we examined the unique contribution of these emotions to the emerging adults’ experiences of S-I in relation to mother and father separately, over and above the relevant demographic variables (age, gender and living arrangement, e.g., Zupančič et al., 2014). We formulated the following hypotheses (H):

H1. The six primary emotions would predict aspects of S-I in relation to parents, over and beyond the emerging adults’ demographic characteristics.

H2. Higher levels of SEEKING would predict higher levels of self-reliance in relation to parents.

H3. Higher level of ANGER would predict higher levels of perceived parental intrusiveness.

H4. Higher levels of both FEAR and SADNESS would predict higher levels of perceived parental intrusiveness and fear of disappointing parents.

H5. Higher levels of both CARE and PLAY would predict connectedness to parents and seeking parental support.

## Method

### Participants and procedure

The participants were 446 emerging adults (58.7% female, 41.3% males), aged from 18 to 30 years (*M* = 21.84 years, *SD* = 2.44). They were mainly students from various faculties of one public university (out of three) in Slovenia. Of the total sample, 233 (52%) were bachelor’s degree students, 102 (22.8%) were master’s degree students, 77 (17.2%) were integrated master’s degree students, 15 (3.3%) were doctoral students, while the remaining 4.2% were not enrolled in education; 17 (3.8%) were employed full time, one (0.2%) was employed part time and one (0.2%) was unemployed; 163 (36.4%) of the participants reported living in their parents’ home, 204 (45.5%) reported to partly live with parents, and 79 (17.6%) reported living out of the parental home.

The study was approved by the National Medical Ethics Commission under code 0120-445/2022/6 and was part of a larger research project which included both molecular genetics and psychological variables. Faculties were contacted through their faculty administration departments which sent e-mails with a short description of the study and an invitation to participate to the enrolled students, along with a link to the online survey. The non-students were acquaintances of the first author of this paper and were invited by individual e-mails. The participants were required to respond to all questions within the online survey to prevent missing data (see the Measures subsection for the only exception).

### Measures

*Demographic characteristics* included questions regarding age (in years and months), gender (an open question; however, qualitative text based responses obtained were only binary, i.e., male and female), current employment/enrollment status (e.g., student, employed full-time, unemployed; all options in the Participants and procedure sub-section), and current living arrangement (with parents, partly living with parents and out of parental home). Then, three self-report questionnaires (one of them in two forms, i.e., in relation to mother and in relation to father) were presented successively, but the order of their presentation was automatically rotated. Participants also had the option to skip the questionnaire related to one of their parents if they did not have a relationship with that parent. As a result, six individuals did not complete the S-I measure in relation to mother, and 22 did not complete it in relation to father.

Brief affective neuroscience personality scales (BANPS; Barrett et al., 2013) consists of 33 items that are rated along a five-point scale (1—strongly disagree, 5—strongly agree). The items represent three positive emotional systems scales, i.e., SEEKING (e.g., *My curiosity drives me to do things.*), PLAY (e.g., *People who know me would say I am a very fun-loving person*.) and CARE (e.g., *I often feel the urge to nurture those closest to me*), as well as three negative emotional systems scales, i.e., FEAR (e.g., *I sometimes cannot stop worrying about my problems.*), SADNESS (e.g., *I often feel lonely.*) and ANGER (e.g., *My friends would probably describe me as hotheaded.*). The six scales have been validated in Slovenia (Glavač and Zupančič, 2024) and showed acceptable psychometric properties—construct validity (e.g., six-factor structure), convergence with the basic personality traits of the BFI (John et al., 1991) and with positive and negative experiences (the SPANE; Diener et al., 2009), internal reliability of the scales and measurement invariance across gender. In the present study, internal reliabilities (αs) were: SEEKING (0.74), FEAR (0.85), ANGER (0.82), SADNESS (0.86), CARE (0.70) and PLAY (0.79).

Individuation test for emerging adults—short (ITEA-S; Komidar et al., 2016) is a 21-item measure scored on a five-point Likert type scale ranging from 1 (completely untrue) to 5 (completely true). The ITEA-S captures five scales (aspects) of S-I specifically in emerging adults and is rated separately in relation to mother and father (these forms differ only grammatically). The scales include Support Seeking (parental support in difficult situations, when having serious problems and/or facing important decisions), Connectedness (to the parent), Perceived Intrusiveness (of the parent), Self-Reliance (in relation to the parent) and Fear of Disappointing (the parent). Both forms have good construct and convergent validity (associations with functional, emotional and conflictual independence, and with measures of self-differentiation), internal consistency and temporal reliability in Slovenian and in Austria, Germany, Portugal, Turkey, and the U.S. samples (Karataş et al., 2019; Komidar et al., 2016, 2021; Nunes et al., 2019; Zupančič and Komidar, 2018). The internal consistencies (αs) in our sample were: Connectedness mother/father (0.82/0.80), Support Seeking (0.91/0.90), Perceived Intrusiveness (0.78/0.85), Self-reliance (0.83/0.87), Fear of Disappointing (0.83/0.87).

## Results

Table 1 presents mean scale scores and standard deviations for the six primary emotions scales and for the five S-I scales in relation to each parent. The table further includes Pearson’s correlation coefficients of emerging adults’ age and gender with the primary emotions scores and with the S-I scores in relation to mother and father, correlations of the primary emotions scores with the S-I scores in relation to each parent, as well as intercorrelations between the primary emotions scores and between the S-I scores. Correlations with living arrangement are not included in the table because they form a categorical variable with three levels. While there is a notable similarity of the pattern of significant correlations between the primary emotions and the aspects of S-I in relation to both parents, more of the associations appear significant in relation to mother (including the relations with demographic variables) and the consistently significant associations also appear slightly stronger in relation to mother than father. To assess potential multicollinearity issues among predictors we used variance inflation factors (VIF) and tolerance statistics, which indicated low to moderate collinearity among the predictors (VIFs = 1.14–2.08; tolerance = 0.48–0.88), suggesting no problematic multicollinearity. To account for within-person dependence between ratings in relation to mother and father, multilevel linear models were conducted with parent modeled as a within-person factor. The results indicated that most primary emotion traits showed comparable associations across parents, suggesting that the overall pattern of findings was largely consistent for mother and father ratings (Supplementary Table S1). However, significant Parent × Emotion interactions emerged for self-reliance (SEEKING and SADNESS) and fear of disappointing (CARE), indicating that associations with these emotional traits differed modestly between S-I ratings in relation to mother and father. Additionally, to control for multiple testing burden, we applied the Benjamini–Hochberg false discovery rate across the 10 regression models that reflect the primary hypotheses within the present study in relation to each parent.

**Table 1:** Descriptive statistics of the six primary emotions scales and the five S-I scales in relation to each parent, correlations among the demographic measures, primary emotions and aspects of S-I, and intercorrelations between the primary emotions and between the aspects of S-I.

Variable	*M*	*SD*	Gender	Age	SEEK	FEAR	ANGER	SAD	CARE	PLAY	SS^a^	CO^a^	PI^a^	SR^a^	FD^a^
SEEK	18.88	3.19	0.19**	0.12*	/	/	/	/	/	/					
FEAR	17.24	4.60	−0.30**	−0.01	0.21**	/	/	/	/	/					
ANGER	15.87	5.25	−0.27**	−0.02	−0.16**	0.41**	/	/	/	/					
SAD	14.92	4.72	−0.23**	0.01	−0.13**	0.66**	0.33**	/	/	/					
CARE	14.74	3.17	−0.23**	0.05	0.09	0.06	0.00	−0.00	/	/					
PLAY	19.97	3.26	0.02	0.02	0.22**	−0.23**	0.03	−0.28**	0.29**	/					
S-I in relation to mother															
SS	17.98	5.23	−0.23**	−0.04	−0.12*	0.12*	0.11*	−0.05	0.15**	0.01	0.36**				
CO	15.51	3.45	−0.03	0.03	−0.01	−0.15**	−0.08	−0.28**	0.22**	0.15**	0.64**	0.41**			
PI	10.98	5.02	0.09	−0.08	−0.03	0.15**	0.09	0.22**	−0.14**	−0.13**	−0.20**	−0.45**	0.38**		
SR	14.22	3.74	0.32**	0.11*	0.30**	−0.20**	−0.13**	−0.10*	−0.11*	0.03	−0.59**	−0.33**	0.18**	0.56**	
FD	9.77	3.48	−0.11*	−0.18**	−0.19**	0.30**	0.11*	0.16**	−0.02	−0.08	0.27**	0.03	0.22**	−0.23**	0.61**
S-I in relation to father															
SS	14.21	5.36	−0.05	−0.06	−0.04	−0.08	−0.04	−0.24**	0.17**	0.08		0.70**	−0.06	−0.62**	0.24**
CO	14.10	3.72	−0.03	0.01	0.05	−0.17**	−0.10*	−0.30**	0.16**	0.15**			−0.25**	−0.41**	0.11**
PI	7.74	3.16	0.12*	−0.06	0.06	0.11*	0.06	0.15**	−0.05	−0.04				0.08	0.17**
SR	15.08	3.56	0.14**	0.06	0.17**	−0.07	−0.07	0.06	−0.08	0.02					−0.17**
FD	9.32	3.64	−0.02	−0.10*	−0.10*	0.23**	0.05	0.06	0.07	0.01					

*N*_(in relation to mother)_ = 440, *N*_(in relation to father)_ = 424. Gender coded 0 for female and 1 for male. S-I = separation-individuation scale scores, SS = support seeking, CO = connectedness, PI = perceived intrusiveness, SR = self-reliance, FD = fear of disappointing, SAD = SADNESS, SEEK = SEEKING. Living arrangement is a categorical variable with three levels (independent, partly living with parents, living with parents), and is therefore not included in the table. *p < 0.05, **p < 0.01.

a Values below the diagonal indicate correlations among aspects of S-I related to mother; values above the diagonal show correlations among the same measures in relation to father. The diagonal entries (rows under the heading S-I in relation to mother) reflect correlations between the corresponding measures in relation to mother and father.

### Predicting separation-individuation in relation to mother

Table 2 shows results of the two-step hierarchical regression analysis predicting the aspects of S-I in relation to mother, with the demographic variables (gender, age and living arrangement) entered in the first block and the six primary emotions scores added simultaneously in the second block (Table S1 in the Supplement presents the confidence intervals for the standardized regression coefficients). The first block predicted statistically significant, but small amounts of the variance (according to Cohen, 1988) in seeking maternal support (6.8%), self-reliance in relation to mother (13.3%) and fear of disappointing her (5.7%). Among the statistically significant single predictors, the male gender was related to higher levels of self-reliance and lower levels of both seeking maternal support and fear of disappointing the mother, younger age associated with higher levels of fear of disappointing the mother, and the participants living partly with parents reported greater levels of self-reliance relative to both emerging adults living with parents and those who moved out of the parental home. When the primary emotions were added in the second step of the analysis, these predictors retained significance (except gender in relation to fear of disappointment), but in addition males scored higher on perceived maternal intrusiveness and the participants who partly moved out of home reported lower levels of seeking maternal support.

**Table 2:** Summary of hierarchical multiple regression predicting the five S-I scores in relation to mother from demographic variables and the six primary emotions scores.

Predictor	Support seeking			Connectedness			Perceived intrusiveness			Self-reliance			Fear of disappointing		
	*B*	SE	*β*	*B*	SE	*β*	*B*	SE	*β*	*B*	SE	*β*	*B*	SE	*β*
Block 1	Δ*R*^2^ = 0.068**			Δ*R*^2^ = 0.008			Δ*R*^2^ = 0.021			Δ*R*^2^ = 0.133**			Δ*R*^2^ = 0.057**		
Gender	−2.32	0.49	−0.23**	−0.17	0.33	−0.03	0.83	0.48	0.08	2.35	0.34	0.32**	−0.66	0.33	−0.10*
Age	0.030	0.11	0.01	0.06	0.07	0.04	−0.14	0.11	−0.07	0.03	0.07	0.02	−0.19	0.07	−0.13**
LA d1	0.405	0.55	0.04	0.51	0.37	0.07	−0.73	0.54	−0.07	0.25	0.38	0.03	0.33	0.37	0.05
LA d2	−1.41	0.76	−0.10	−0.05	0.52	−0.01	−0.88	0.75	−0.07	1.79	0.53	0.18**	−0.94	0.51	−0.10
Block 2	Δ*R*^2^ = 0.052**			Δ*R*^2^ = 0.129**			Δ*R*^2^ = 0.075**			Δ*R*^2^ = 0.071**			Δ*R*^2^ = 0.098**		
Gender	−1.88	0.52	–0.18**	0.29	0.34	–0.04	1.32	0.51	0.13**	1.75	0.36	0.24**	−0.08	0.34	–0.01
Age	0.040	0.11	0.02	0.06	0.07	0.04	−0.15	0.10	–0.07	−0.00	0.07	–0.00	−0.18	0.07	–0.12*
LA d1	0.202	0.54	0.02	0.43	0.35	0.06	−0.72	0.52	–0.07	0.42	0.37	0.06	0.21	0.35	0.03
LA d2	−1.47	0.75	–0.11	−0.11	0.49	–0.01	−0.84	0.73	–0.06	1.84	0.51	0.19**	−0.08	0.49	–0.11*
SEEK	−0.10	0.08	−0.06	−0.04	0.05	−0.04	0.02	0.08	0.01	0.28	0.05	0.24**	−0.12	0.05	−0.11*
FEAR	0.21	0.07	0.18**	0.02	0.05	0.03	0.04	0.07	0.04	−0.10	0.05	−0.12*	0.26	0.05	0.34**
ANGER	0.06	0.05	0.06	−0.00	0.03	−0.01	0.04	0.05	0.04	−0.00	0.04	−0.01	−0.03	0.03	−0.04
SAD	−0.28	0.07	−0.25**	−0.23	0.05	−0.31**	0.21	0.07	0.20**	0.05	0.05	0.06	−0.04	0.05	−0.06
CARE	0.18	0.08	0.11*	0.21	0.05	0.20**	−0.14	0.08	−0.09	−0.09	0.06	−0.07	−0.05	0.05	−0.04
PLAY	−0.03	0.08	−0.02	0.03	0.05	0.03	−0.07	0.08	−0.05	−0.02	0.06	−0.02	0.04	0.05	0.03
Total *R*^2^	*R*^2^ = 0.119			*R*^2^ = 0.136			*R*^2^ = 0.136			*R*^2^ = 0.204			*R*^2^ = 0.155		
Adj *R*^2^	*R*^2^ = 0.099			*R*^2^ = 0.116			*R*^2^ = 0.116			*R*^2^ = 0.185			*R*^2^ = 0.136		

*N* = 440. Gender coded 0 for females and 1 for males. SEEK = SEEKING, SAD = SADNESS. LA = living arrangement (LA d1 coded for living with parents; LA d2 coded for partly living with parents; living independently coded 0 for both dummy variables). *p < 0.05, **p < 0.01.

According to our first hypothesis, the block of six primary emotions provided a significant increment in the variance explained across the aspects of S-I, over and above the block of demographic variables, i.e., additional 5.2% of the variance in seeking maternal support, 12.9% in feelings of connectedness to mother, 7.5% in perceived maternal intrusiveness, 7.1% in self-reliance, and 9.8% in fear of disappointing the mother. Among the significant single predictors, higher levels of FEAR (*β* = 0.18) and CARE (*β* = 0.11; in line with H5), but lower levels of SADNESS (*β* = −0.25) associated with higher levels of seeking maternal support. Likewise, lower levels of SADNESS (*β* = −0.31) and higher levels of CARE (*β* = 0.20; in support to H5) were related to stronger connectedness to mother. In contrast to support seeking and connectedness, higher levels of SADNESS (*β* = 0.20) associated with higher levels of perceived maternal intrusiveness, supporting H4. Individual differences in SEEKING and FEAR contributed to the remaining two aspects of S-I in relation to mother, but in the opposite directions. While higher levels of SEEKING (*β* = 0.24; consistent with H2) and lower levels of FEAR (*β* = −0.12) correlated with greater levels of self-reliance, lower levels of SEEKING (*β* = −0.11) and higher levels of FEAR (*β* = 0.34; in line with H4) were related to higher levels of fear of disappointing the mother.

In sum, the two blocks of predictors statistically significantly accounted for 9.5 to 20.4% of the variance in S-I scores in relation to mother. As recommended by Cohen (1988), these values represent small portions of variance explained in perceived maternal intrusiveness and seeking maternal support, and moderate portions of variance in explaining connectedness to mother, fear of disappointing the mother, and self-reliance within the relationship.

### Predicting separation-individuation in relation to father

Table 3 shows results of the two-step hierarchical regression predicting the aspects of S-I in relation to father (Supplementary Table S1 presents the confidence intervals for the standardized regression coefficients). We performed the same analysis to the one predicting S-I in relation to mother. The block of demographic variables explained statistically significant, but small amounts of the variance in perceived paternal intrusiveness (3.9%), self-reliance in relation to father (3.3%) and fear of disappointing him (1.8%), with males scoring significantly higher in perceived paternal intrusiveness and self-reliance, and emerging adults living partly with parents reporting on lower levels of perceived paternal intrusiveness and higher levels of self-reliance (relative to those living with parents or moved out of parental home). When the primary emotions were added in the second step of the analysis, these predictors retained significance.

**Table 3:** Summary of hierarchical multiple regression predicting the five S-I scores in relation to father from demographic variables and the six primary emotions scores.

Predictor	Support seeking			Connectedness			Perceived intrusiveness			Self-reliance			Fear of disappointing		
	*B*	SE	*β*	*B*	SE	*β*	*B*	SE	*β*	*B*	SE	*β*	*B*	SE	*β*
Block 1	Δ*R*^2^ = 0.008			Δ*R*^2^ = 0.007			Δ*R*^2^ = 0.039**			Δ*R*^2^ = 0.033**			Δ*R*^2^ = 0.018*		
Gender	−0.53	0.52	−0.05	−0.17	0.36	−0.02	0.70	0.30	0.11*	1.04	0.34	0.15**	−0.02	0.35	−0.00
Age	−0.09	0.16	−0.04	−0.06	0.08	−0.03	0.02	0.07	−0.02	0.02	0.08	0.02	−0.12	0.08	−0.08
LA d1	−0.21	0.59	−0.02	0.55	0.41	0.07	−0.64	0.34	−0.10	0.45	0.38	0.06	0.64	0.40	0.09
LA d2	−0.72	0.83	−0.05	0.66	0.57	0.07	−1.35	0.48	−0.16**	1.12	0.54	0.12*	−0.17	0.56	−0.02
Block 2	Δ*R*^2^ = 0.097**			Δ*R*^2^ = 0.106**			Δ*R*^2^ = 0.042**			Δ*R*^2^ = 0.048**			Δ*R*^2^ = 0.081**		
Gender	−0.53	0.55	–0.05	−0.52	0.38	–0.07	0.99	0.33	0.16**	0.75	0.37	0.11*	0.57	0.37	0.08
Age	−0.07	0.11	–0.03	−0.04	0.08	–0.03	−0.04	0.07	–0.03	0.00	0.08	0.00	−0.13	0.08	–0.09
LA d1	−0.34	0.56	–0.03	0.53	0.38	0.07	−0.61	0.34	–0.10	0.57	0.38	0.08	0.58	0.38	0.08
LA d2	−0.77	0.79	–0.03	0.64	0.54	0.06	−1.36	0.47	–0.16**	1.14	0.53	0.12*	−0.22	0.54	–0.02
SEEK	−0.09	0.08	−0.05	0.02	0.06	0.02	0.07	0.05	0.07	0.15	0.06	0.14**	−0.07	0.06	−0.06
FEAR	0.12	0.08	0.10	0.03	0.05	0.04	0.05	0.05	0.07	−0.09	0.05	−0.12	0.29	0.05	0.36**
ANGER	0.00	0.05	0.00	−0.03	0.04	−0.04	0.02	0.03	0.04	−0.02	0.04	−0.04	−0.04	0.04	−0.05
SAD	−0.37	0.07	−0.33**	−0.26	0.05	−0.33**	0.09	0.04	0.14*	0.15	0.05	0.20**	−0.10	0.05	−0.14*
CARE	0.27	0.09	0.16**	0.14	0.06	0.12*	−0.02	0.05	−0.02	−0.09	0.06	−0.08	0.07	0.06	0.06
PLAY	−0.04	0.09	−0.02	0.04	0.06	0.03	0.01	0.05	0.01	0.05	0.06	0.05	0.06	0.06	0.05
Total *R*^2^	*R*^2^ = 0.104			*R*^2^ = 0.121			*R*^2^ = 0.081			*R*^2^ = 0.081			*R*^2^ = 0.099		
Adj *R*^2^	*R*^2^ = 0.083			*R*^2^ = 0.106			*R*^2^ = 0.059			*R*^2^ = 0.059			*R*^2^ = 0.077		

*N* = 424. Gender coded 0 for females and 1 for males. SEEK = SEEKING, SAD = SADNESS. LA = living arrangement (LA d1 coded for living with parents; LA d2 coded for partly living with parents; living independently coded 0 for both dummy variables). *p < 0.05, **p < 0.01.

In agreement with our first hypothesis, the block of primary emotions scale scores statistically significantly improved the portions of variance explained across the aspects of S-I by 9.7% (seeking paternal support), 10.6% (connectedness to father), 4.2% (paternal intrusiveness), 4.8% (self-reliance within the relationship), and 8.1% (fear of disappointing the father), over and beyond the block of demographic variables. Among the significant single predictors, lower levels of SADNESS and higher levels of CARE (consistent with H5) were related with greater levels of both seeking paternal support (*β* = −0.33 and *β* = 0.16, respectively) and connectedness to father (*β* = −0.33 and *β* = 0.12, respectively), whereas higher levels of SADNESS (*β* = 0.14) associated with higher levels of both perceived paternal intrusiveness (as proposed by H4) and self-reliance (*β* = 0.20), but with lower levels of fear of disappointing the father (*β* = −0.14; contrasting H4). Furthermore, emerging adults scoring higher in SEEKING reported higher levels of self-reliance (*β* = 0.14; consistent with H2) and those scoring higher in FEAR exhibited higher levels of fear of disappointing the father (*β* = 0.36, aligning with H4).

Jointly, the two blocks of predictors accounted for statistically significant, but small portions of the variance explained in the aspects of S-I within the emerging adult-father relationship—8.1% in both perceived intrusiveness and self-reliance, 9.9% in fear of disappointing, 10.4% in support seeking, and 12.1% in feelings of connectedness to father.

### Summary of the predictive relations between primary emotions and aspects of separation–individuation

The results of both predictive analyses suggested full support to H1 and H2, partial support to H4 and H5, and no support to H3. The primary emotions of ANGER and PLAY did not relate statistically significantly to any of the aspects of S-I in relation to either mother or father, seven significant single predictions were consistent across parents and four were specific to each parent. Specific associations of the primary emotions with the aspects of S-I that were not expected were also found statistically significant. These were: higher SEEKING—lower levels of fear of disappointing the mother, higher FEAR—higher levels of seeking maternal support and lower levels of self-reliance, and higher SADNESS—lower levels of seeking maternal/paternal support and lower levels of connectedness to mother/father.

## Discussion

The present study investigated the role of primary emotions, conceptualized within the framework of Affective Neuroscience Theory (ANT), in shaping emerging adults’ experiences of separation-individuation (S-I) in relation to parents, over and above previously documented age, gender and living arrangement of young people (e.g., Komidar et al., 2016; Kavčič and Zupančič, 2019; Saraiva and Matos, 2012; Sneed et al., 2006). The block of these demographic characteristics indeed showed significant, but modest effects on limited number of aspects of S-I, particularly indicating that males tended to report greater self-reliance and lower support seeking, reflecting possible gendered expectations in autonomy development (Eagly, 1987; Geuzaine et al., 2000). Relative to individuals who co-resided with parents and moved out of their family home, those partly living with parents also felt more self-reliant and less likely to seek parental support; this suggests that gradually taking up responsibilities of an independent household may promote healthy S-I in the Slovenian cultural context, characterized by normatively strong reliance on one’s family and expected prolonged dependence on all kinds of parental support (Kuhar and Reiter, 2014). More importantly, the block of six primary emotions significantly improved the variance explained in all S-I measures beyond the demographic variables.

Our findings offer novel support for the proposition that individual differences in primary emotional systems contribute uniquely and meaningfully to the understanding of how emerging adults navigate the process of S-I within their relationships with parents. Nevertheless, the primary emotions showed modest associations with the S-I outcomes and the regression models explained moderate proportions of the variance, highlighting that while the biologically based emotional systems contribute to the S-I dynamics, these processes are likely also shaped by a wide array of contextual, relational, and developmental factors.

More nuanced findings of this study lie in the comparison of patterns of association between the primary emotions across the aspects of S-I in relation to mother versus father. While the general pattern of associations was substantially consistent—suggesting that primary emotions are broadly relevant across both parent–child relationships—some differences emerged in the magnitude, direction, and specificity of the predictions. First, we discuss the unique contribution of each primary emotion in relation to the aspects of S-I.

### Primary emotions as predictors of separation-individuation

As hypothesized, SEEKING was positively associated with self-reliance in relation to both parents, substantiating its proposed role in facilitating autonomy and exploration. This is consistent with research linking SEEKING to the basic personality trait of openness (Davis and Panksepp, 2011; Marengo et al., 2021) that are suggested to share a common neurobiological basis (Montag and Davis, 2018). The SEEKING system, linked to the dopaminergic system, drives motivation and exploration (Panksepp, 1998). Individuals high in SEEKING and openness tend to be curious, inquisitive, and motivated to explore beyond the family environment and thus, likely gain experiences that foster competence and self-reliance. Our finding aligns with literature suggesting that open and exploratory traits promote independent functioning during emerging adulthood (Luyckx et al., 2012; Poredoš, 2020; Zupančič and Kavčič, 2014). In addition, we revealed an inverse association between SEEKING and fear of disappointing the mother, implying that higher intrinsic motivation to explore and assert one’s own goals may buffer against concerns of parental disapproval. These outcomes stress an important role of SEEKING in supporting autonomy within close relationships.

Congruent with our assumption, FEAR was positively associated with fear of disappointing both parents when their expectations and norms were not met by the emerging adult child. These results reinforce the conceptualization of FEAR as a dispositional correlate of neuroticism and behavioral inhibition (Barlow et al., 2014; Gray, 1987; Montag and Panksepp, 2017), which may interfere with the confidence required to achieve a healthy balance between psychological separation from caregivers and maintaining relatedness to them. Specifically, the heightened sensitivity to threat in high-FEAR individuals (Britton et al., 2011; Sagliano et al., 2014) may augment their concern with meeting parental expectations, thereby hindering S-I. Aside from the hypothesized predictions, higher levels of self-reported FEAR among young people were associated with both lower levels of their self-reliance in relation to mother and stronger tendencies to turn to her for opinion, advice and help in difficult situations and/or in making important life decisions. FEAR as an index of neurotic vulnerability and anxiety-prone traits (Barlow et al., 2014; Caspi and Shiner, 2006) also showed links to stronger dependency on parents and parental emotional over-involvement in emerging adult children’s lives (Kins et al., 2013; Poredoš, 2020; Zupančič and Kavčič, 2014). Taken together, the consistency of FEAR in explaining these outcomes underscores the emotional cost of S-I in emerging adults with heightened threat sensitivity, who may be more attuned to possible relational ruptures during the second S-I.

SADNESS revealed complex associations with the aspects of S-I. While higher levels of SADNESS were associated with greater perceptions of parental intrusiveness (as proposed) and was inversely associated with fear of disappointing the father (opposing our hypothesis), it also showed negative relations to both seeking parental support and feelings of connectedness to parents. These mixed findings suggest that individuals with higher dispositional SADNESS may perceive greater emotional distance from parents, possibly due to their increased emotional sensitivity and/or attachment avoidance. Indeed, Kins et al. (2013) found that dysfunctional independence within parent–child relationships was moderately related to emerging adults’ discomfort with closeness and reliance on others. Alternatively, the withdrawal tendencies of individuals high in SADNESS map onto the trait of neuroticism (Caspi and Shiner, 2006) that also mirrors social inhibition in close relationships (Karataş et al., 2019; Zupančič et al., 2011; Zupančič and Kavčič, 2014). This further suggests that the propensity toward SADNESS may be linked to poor engagement of emerging adults with parental figures, reducing perceived expectations of receiving parental support and promoting a compensatory form of independence.

The prosocial emotion of CARE—considered as the biological basis of agreeableness (Davis and Montag, 2019)—was associated with higher levels of feelings of connectedness to parents and seeking parental support in particularly challenging and/or difficult situations. This outcome aligns with the conceptualization of the CARE system as an evolutionary mechanism promoting affiliation, social approach and bonding (e.g., Davis and Panksepp, 2011; Panksepp, 1998). The mechanism is linked to the oxytocin and opioid neurobiological pathways (Panksepp, 1998) and likely facilitates empathy, prosocial behavior, social communication and affection, and security of attachment (Jones et al., 2015; Panksepp, 1998). Furthermore, the characteristics of the CARE system are subsumed by the trait of agreeableness (e.g., Glavač and Zupančič, 2024; Marengo et al., 2021), which promotes favorable and stable relationships with significant others, such as warmth in parent-adolescent relationships (De Haan et al., 2012), and internal representations of mutually respectful, understanding, trustful and supportive relationships with parents (Poredoš, 2020; Zupančič and Kavčič, 2014).

In contrast to our hypotheses, ANGER and PLAY had no significant role in perceived parental intrusiveness and in aspects of relatedness (connectedness to parents and seeking parental support), respectively. Considering ANGER, our outcomes do not agree to the expectations based on the notable associations of this primary system with low agreeableness and interpersonal conflict (Bettencourt et al., 2006; Glavač and Zupančič, 2024; Marengo et al., 2021), and of low agreeableness with perceived parental intrusiveness in emerging adulthood (Poredoš, 2020; Zupančič and Kavčič, 2014). The non-significant relation between ANGER and the respective aspect of S-I in the present study might reflect the intrapsychic nature of the S-I, where young people may internalize perceptions of parental intrusiveness more than overtly externalize anger toward parental control, excessive care and undesirable intrusion into their privacy. Emerging adults may experience parental intrusiveness as an internal tension rather than responding with explicit defensive reactions. Additionally, socialization processes and cultural norms often discourage the open expression of anger toward parents, potentially leading to suppression or transformation of anger into less overt emotional responses (Eisenberg et al., 1998; Matsumoto et al., 2008). Consistently, research on psychological control suggests that emotion regulation plays a key role in the association between controlling parenting and aggressive responses (Cui et al., 2014), highlighting the importance of regulatory processes in shaping how anger is experienced and expressed. From this perspective, perceived intrusiveness may be managed through withdrawal, compliance, or internal distress (Barber, 1996; Soenens and Vansteenkiste, 2010). Furthermore, the trait-based self-report measures of ANGER may capture general dispositional irritability but not situation-specific or relational expressions of anger that arise in parent–child interactions, highlighting the importance of distinguishing between trait and state emotional processes. We also cannot rule out the possibility that ANGER may be, relative to the four previously discussed emotions, less central in the process of establishing a comfortable balance between autonomy and relatedness to parents during emerging adulthood.

Similar may apply in relation to PLAY. This emotional system presumably represents the subcortical foundation of extraversion (Davis and Panksepp, 2011; Montag and Panksepp, 2017) which associates with positive parent–child relationships (e.g., De Haan et al., 2012; Denissen et al., 2009), connectedness to parents and seeking their support (Poredoš, 2020; Zupančič and Kavčič, 2014). However, individual differences in PLAY (enjoyment, liveliness, eagerness and social approach) suggested no association with the two aspects of S-I. Perhaps these characteristics relate to one’s tendencies toward extensive rather than emotionally intensive social engagement and to an establishment of broader but less emotionally involved social ties rather than to maintenance of emotionally close relationships. The null findings in our sample may be also due to the shared variance of PLAY with other primary emotions (CARE and SADNESS) that were predictive of the respective aspects of S-I. Relatedly, the primary emotions showed moderate intercorrelations, particularly between FEAR and SADNESS, indicating shared variance among the affective dimensions. Although multicollinearity diagnostics were within acceptable ranges, such overlap may attenuate unique regression coefficients and contribute to non-significant effects for theoretically relevant predictors such as PLAY and ANGER.

### Consistent and differential predictive patterns across parental figures

The two blocks of variables accounted for somewhat more variance in aspects of S-I in relation to mother than father, and the additional variance explained by the block of primary emotions was also greater (except for support seeking). Nevertheless, the patterns of association between individual emotions and aspects of S-I were generally more similar than different across parental figures. A key consistency was the association of higher CARE and lower SADNESS with both stronger parent-emerging adult child emotional ties (connectedness) and seeking parental support when coping with difficult life situations. In contrast, higher SADNESS consistently associated with stronger perceptions of parental over-concern, poor respect for the emerging adult child’s privacy and feelings of being psychologically controlled by the parents (intrusiveness). These findings converge with the view that prosocial (CARE) and internalizing (SADNESS) emotional systems likely reflect universal mechanisms of attachment and interpersonal approach or withdrawal (Brumariu and Kerns, 2010; Sroufe, 2005). Further, lower FEAR and higher SEEKING were associated with less fear of disappointing the parents and more self-reliance in relation to both parental figures, respectively, which suggests that these emotions may be broadly linked to autonomy development and anxiety-based emotional regulation in the parent-emerging adult relationships.

Along with these consistent patterns of links across the parental figures, we identified a few parent-specific associations of emerging adults’ self-perceived expression of primary emotions and representations of self within their relationships with parents. SEEKING was negatively related to fear of disappointing the mother, suggesting that autonomy-oriented individuals may feel more confident asserting independence not only without heightened but actually reduced anxiety over maternal disapproval. In contrast, higher levels of FEAR among young people were associated with both lower levels of their self-reliance in relation to mother and stronger tendencies to turn to her for opinion, advice and help. Findings suggest that young people’s relationships with their mother tend to be more mutually involved in day-to-day interactions, emotionally closer, confidential, cooperative and symmetrical (Buhl, 2008; Collins and Laursen, 2004; Lye, 1996; Proulx and Helms, 2008) than father-child relationships. Fear-prone individuals may feel less self-assured in the process of establishing adult autonomy and may have a stronger need to rely on the most trustful, caring and reliable figure, particularly in personally difficult situations, challenging transitions or turning points in their life, either for consolation, emotional support, advice, opinion or help. Considering the subtle differences in mother– and father–child relationships just mentioned, high-FEAR emerging adults would be more likely to turn to their mother first for emotional support.

Another parent-specific pattern of associations were somewhat divergent for SADNESS in relation to father, as it was associated with higher self-reliance and lower fear of disappointing the father. Higher SADNESS is correlated with an anxious attachment style and inversely correlated with a secure attachment style (Fuchshuber et al., 2019). More specifically, individuals with an avoidant attachment style have been found to have a history of their support-seeking needs not being met by caregivers (likely triggering experiences of separation-distress, i.e., SADNESS) (Richardson et al., 2023). Such a history predisposes them to minimize outward signs of distress (Dozier and Kobak, 1992; Richardson et al., 2023) and coincides with a profile of compulsive self-reliance (Mikulincer and Shaver, 2004). Our results suggest that individuals high in SADNESS may experience emotional distance or presumably poorly met attachment needs more acutely with their fathers. As a result, they are likely to adopt compensatory (more independent) strategies of resolving the autonomy-relatedness issues within the father–child relationships. In this case, self-reliance may emerge as a defensive adaptation rather than an indicator of healthy S-I. Reflecting this point, research on S-I in emerging adulthood has shown that high self-reliance, combined with aspects of dysfunctional separation-individuation, such as low support-seeking and high fear of disappointing the parent, implies that overexpressed self-reliance could serve as a defensive adaptation (Kavčič and Zupančič, 2019).

Although separate regression analyses suggested some differences in the regression coefficients across parents, multilevel models indicated that most associations between the primary emotions and aspects of S-I did not differ significantly across parent–child relationships. However, a few of these differences across parental figures suggest the importance of considering each parent–child dyad as a unique emotional ecology, shaped by distinct histories, relational roles, and cultural expectations that modulate how biologically rooted emotional traits influence the resolution of the developmental task of S-I in relation to parents.

### Limitations and future directions

A notable limitation of our study that prevents generalizations to the broader population of emerging adults is the convenience sampling, resulting in an over-representation of younger emerging adults (below 24 years) and students who represent approximately half of all emerging adults, aged from 18 to 24 years, in Slovenia (SURS, 2024). Therefore, findings may not generalize to the broader emerging adult population, particularly those not in education or employment. Likewise, our sample was slightly over-represented by females. The correlational nature of the study design also does not allow any conclusions about the directionality of the relations revealed. Feelings of connectedness within the parent–child relationships may, for example, augment emerging adults’ expression of the CARE system, not only the other way around. Thus, cross-lagged panel designs, relying on longitudinal data and controlling for temporal stability of the constructs under investigation, are warranted. As our data were entirely based on emerging adults’ self-reports, the associations obtained may have been somewhat inflated due to the shared method variance. Taking on a multiple-informant approach in the respective area of investigation may offer a better, or at least more balanced insight into the links between the primary emotions and aspects of S-I.

To this point subsequent research using state-based or observational approaches may further clarify the situational role of the primary emotions in aspects of S-I. Future research would also greatly benefit from more complex designs that account for the interplay of both emerging adults’ and their parents’ emotionally-based characteristics (e.g., Poredoš, 2020), parenting styles (Wolfradt et al., 2003; Yazdani and Daryei, 2016) or dimensions of parenting, such as autonomy support and psychological control (Pérez et al., 2021; Romm et al., 2019; Soenens and Beyers, 2012), various qualities of parent–child relationships (e.g., Sneed et al., 2006), and the developmental history of these relationships. Furthermore, because the present study did not include the established broad-band personality traits, future research should examine primary emotions alongside the basic personality traits (e.g., the Big Five) to directly test for potential incremental validity of the primary emotions. Finally, while the ANT framework offers a compelling neurobiological account of emotional foundations in personality, research is still needed to refine how these systems manifest in everyday interpersonal contexts and developmental transitions.

## Conclusion

This study offers a new perspective to supplement traditional views of S-I that emphasize caregiving history and relational dynamics by integrating individual differences in emotional predispositions into the model. Our findings lend empirical support to the theoretical view that along with a variety of relational and contextual factors the primary emotions also represent a meaningful biological and emotional substrate associated with emerging adults’ capacity to simultaneously maintain connectedness to parents while asserting psychological autonomy. Although the variance explained by the primary emotions, over and beyond the block of relevant demographic variables, was relatively small for some aspects of S-I, the results indicate that primary emotions represent one of several contributing factors associated with individual differences in S-I. Specifically, four of the six primary emotions, as conceptualized within the ANT, significantly and uniquely related to all included aspects of S-I (connectedness to parents, seeking parental support, perceived parental intrusiveness, self-reliance, and fear of disappointing parents) in emerging adulthood. These outcomes extend current models of S-I by integrating neuro-biologically grounded emotional systems and suggest that emotionally-based predispositions, particularly SEEKING, FEAR, SADNESS, and CARE, form a meaningful part of how young people establish autonomous yet connected identities within their parent–child relationships. While the primary emotions as single predictors consistently contributed to the same aspects of S-I across both parental relationships, a few associations specific to each parent were also suggested. This highlights the importance of differentiating between relationships with mother and father, as these appear to be shaped by partially distinct emotional and relational dynamics. Such differentiation is essential for both theoretical refinement and practical application in developmental psychology and family therapy settings. Overall, our findings contribute to ongoing efforts to integrate biologically informed perspectives with relational models of S-I, while underscoring that emotional traits represent an important component within the complex developmental processes shaping S-I in emerging adulthood.

## Data Availability

The raw data supporting the conclusions of this article will be made available by the authors, without undue reservation.

## Footnotes

1 The names of these primary emotions are capitalized to distinguish them from emotion terms used in common vernacular.
