# Supplementary material for: The role of primary emotions in separation-individuation during emerging adulthood
Source: Front Psychol. 2026 Apr 14;17:1675495. doi: 10.3389/fpsyg.2026.1675495 (PMC13120976; doi:10.3389/fpsyg.2026.1675495)
Supplement: Supplementary file 1 [file Table_1.docx]

**Supplementary material**

**Table S1.** Confidence intervals for standardized regression coefficients predicting aspects of separation-individuation in relation to mother with demographic variables and the primary emotions

| Predictor | Support seeking | Connectedness | Perceived intrusiveness | Self- reliance | Fear of disappointing |
| --- | --- | --- | --- | --- | --- |
|  | β 95% | β 95% | β 95% | β 95% | β 95% |
| Block 1 |  |  |  |  |  |
| Gender | -3.28 – -1.37 | -.82 – .48 | -.11 – 1.77 | 1.69 – 3.01 | -1.30 – -.02 |
| Age | -1.81 – .242 | -.09 – .20 | -.35 – .07 | -.12 – .18 | -.33 – -.04 |
| LA d1 | -.67 – 1.48 | -.22 – 1.24 | -1.78 – .33 | -.49 – .99 | -.39 – 1.04 |
| LA d2 | -2.90 – .09 | -1.07 – .97 | -2.35 – .60 | .76 – 2.28 | -1.94 – .06 |
| Block 2 |  |  |  |  |  |
| Spol | -2.90 – -.85 | -.96 – .38 | .32 – 2.32 | 1.05 – 2.45 | -.75 – .59 |
| Starost | -.17 – .25 | -.08 – .20 | -.35 – .06 | -.15 – .14 | -.31 – -.04 |
| LA d1 | -.85 – 1.26 | -.26 – 1.12 | -1.75 – .31 | -.30 – 1.14 | -.47 – .90 |
| LA d2 | -2.94 – -.01 | -1.06 – .85 | -2.26 – .59 | .84 – 2.83 | -1.93 – -,02 |
| SEEKING | -.26 – .05 | -.15 – .06 | -.13 – .17 | .18 – .39 | -.22 – -.02 |
| FEAR | .06 – .35 | -.07 – .12 | -.10 – .19 | -.20 – -.01 | .16 – .35 |
| ANGER | -.04 – .16 | -.07 – .06 | -.06 – .14 | -.07 – .07 | -.09 – .04 |
| SADNESS | -.41 – -.14 | -.32 – -.14 | .08 – .35 | -.04 – .14 | -.13 – .05 |
| CARE | .02 – .34 | .11 – .32 | -.29 – .02 | -.20 – .02 | -.15 – .06 |
| PLAY | -.19 – .13 | -.08 – .14 | -.23 – .09 | -.13 – .09 | -.07 – .14 |

**Table S2.** Confidence intervals for standardized regression coefficients predicting aspects of separation-individuation in relation to father with demographic variables and the primary emotions

| Predictor | Support seeking | Connectedness | Perceived intrusiveness | | Self-reliance | Fear of disappointing |
| --- | --- | --- | --- | --- | --- | --- |
|  | β 95% | β 95% | β 95% | β 95% | | β 95% |
| Block 1 |  |  |  |  | |  |
| Gender | -1.57 – .49 | -.88 – .54 | .099 – 1.29 | .36 – 1.71 | | .71 – .68 |
| Age | -.32 – .14 | -.21 – .11 | -.15 – .11 | -.13 – .17 | | -.28 – .03 |
| LA d1 | -1.36 – .94 | -.25 – 1.35 | -1.30 – .03 | -.30 – 1.21 | | -.13 – 1.42 |
| LA d2 | -2.34 – .91 | -.47 – 1.78 | -2.29 – -.41 | .05 – 2.18 | | -1.26 – .93 |
| Block 2 |  |  |  |  | |  |
| Gender | -1.61 – .55 | -1.25 – .22 | .34 – 1.63 | .03 – 1.48 | | -.16 – 1.31 |
| Age | -.29 – .15 | -.19 – .11 | -.17 – .09 | -.14 – .15 | | -.28 – .02 |
| LA d1 | -1.44 – .77 | -.22 – 1.29 | -1.27 – .05 | -.17 – 1.32 | | -.18 – 1.33 |
| LA d2 | -2.32 – .79 | -.43 – 1.70 | -2.29 – -.43 | .09 – 2.18 | | -1.28 – .83 |
| SEEKING | -.25 – .07 | -.09 – .13 | -.03 – .17 | .04 – .26 | | -.18 – .05 |
| FEAR | -.04 – .27 | -.07 – .14 | -.04 – .14 | -.20 – .01 | | .18 – .39 |
| ANGER | -.11 – .11 | -.10 – .05 | -.04 – .09 | -.10 – .05 | | -.11 – .04 |
| SADNESS | -.51 – -.23 | -.35 – -.16 | .01 – .18 | .06 – .25 | | -.20 – -.01 |
| CARE | .11 – .44 | .03 – .26 | -.12 – .08 | -.21 – .02 | | -.05 – .18 |
| PLAY | -.21 – .13 | -.08 – .15 | .09 – .11 | -.06 – .16 | | -.06 – .17 |

**Table S3.** Multilevel Linear Regression Models Predicting Separation–Individuation Dimensions From Primary Emotions and Parent

| Predictor | Support Seeking | | | Connectedness | | | Perceived Intrusiveness | | | Self-Reliance | | | Fear of Disappointing | | |
| --- | --- | --- | --- | --- | --- | --- | --- | --- | --- | --- | --- | --- | --- | --- | --- |
|  | B | SE | *p* | B | SE | *P* | B | SE | *P* | B | SE | *p* | B | SE | *p* |
|  |  | | |  | | |  | | |  | | |  | | |
| Intercept | 17.95 | .24 | < .001 | 15.50 | .16 | < .001 | 10.99 | .02 | <.001 | 14.23 | .17 | < .001 | 9.79 | .16 | <.001 |
| Parent | -3.75 | .28 | < .001 | -1.39 | .19 | < .001 | -3.26 | .23 | <.001 | .88 | .16 | < .001 | -.44 | .15 | .003 |
| SEEKING | -0.16 | .08 | .041 | -.05 | .05 | .32 | .04 | .06 | .551 | .34 | .06 | < .001 | -.16 | .05 | .004 |
| FEAR | .22 | .08 | .003 | .03 | .05 | .57 | .02 | .06 | .704 | -.12 | .05 | .025 | .25 | .05 | <.001 |
| ANGER | .10 .05 .066 | | | .01 .03 .92 | | | .01 .04 .744 | | | -.03 .04 .339 | | | -.03 .04 .458 | | |
| SADNESS | -.25 .07 < .001 | | | -.22 .05 < .001 | | | .20 .06 <.001 | | | .04 .05 .457 | | | -.04 .05 .389 | | |
| CARE | .25 .08 .002 | | | .23 .05 < .001 | | | -.20 .07 .002 | | | -.14 .06 .010 | | | -.05 .05 .379 | | |
| PLAY | -.03 .08 .688 | | | .03 .06 .632 | | | -.06 .07 .351 | | | -.02 .06 .771 | | | .03 .06 .584 | | |
| Parent × SEEKING | .06 .09 .544 | | | .06 .06 .366 | | | .04 .08 .612 | | | -.17 .05 **.001***** | | | .07 .05 .174 | | |
| Parent × FEAR | -.10 | .09 | .275 | .01 | .06 | .850 | -.00 | .07 | .989 | .01 | .05 | .875 | .02 | .05 | .662 |
| Parent × ANGER | -.08 | .06 | .172 | -.01 | .04 | .742 | -.00 | .05 | .848 | -.00 | .04 | .956 | -.02 | .03 | .476 |
| Parent × SADNESS | -.11 | .08 | .194 | -.03 | .06 | .628 | -.11 | .07 | .093 | .11 | .05 | **.019*** | -.06 | .04 | .161 |
| Parent × CARE | .04 | .09 | .702 | -.07 | .06 | .278 | .14 | .08 | .069 | .04 | .05 | .506 | .10 | .05 | **.049*** |
| Parent × PLAY | .01 | .10 | .964 | .15 | .07 | .817 | .07 | .08 | .370 | .06 | .06 | .258 | .03 | .05 | .617 |

*Notes. p* < .05, p** < .01, mother = 0, father = 1.*
